# Cranial radiation therapy with hippocampus avoidance in lung cancer treatment: systematic review and meta-analysis

**DOI:** 10.3389/fonc.2023.1268754

**Published:** 2023-10-23

**Authors:** Yue Zheng, Liting You, Baijie Feng, Min Tang, Feifei Na

**Affiliations:** ^1^ Division of Thoracic Tumor Multimodality Treatment and Department of Radiation Oncology, Cancer Center, West China Hospital, Sichuan University, Chengdu, Sichuan, China; ^2^ Department of Laboratory Medicine, West China Hospital, Sichuan University, Chengdu, Sichuan, China; ^3^ West China School of Medicine, Sichuan University, Chengdu, Sichuan, China

**Keywords:** hippocampus avoidance, prophylactic cranial irradiation, whole brain radiotherapy, neurocognitive function, lung cancer

## Abstract

**Background:**

The role of cranial radiation therapy with hippocampus avoidance (HA-CRT) in neurocognitive function (NCF), brain metastasis (BM), and overall survival (OS) in lung cancer remains unclear.

**Methods:**

A meta-analysis was conducted to evaluate the impact of HA-CRT in lung cancer. Data from studies on hippocampal-avoidance prophylactic cranial irradiation (HA-PCI) and whole brain radiotherapy (HA-WBRT) were pooled.

**Results:**

A total of 14 studies, including 5 randomized controlled trials, were included. The focus of NCF was mainly *via* the Hopkins Verbal Learning Test—Revised or the Free and Cued Selective Reminding Test. At 6 months post-radiotherapy, the pooled proportion of participants with decline in the performance of total recall, delayed recall, and discrimination in neurocognitive tests were 0.22 (95% CI 0.15, 0.29), 0.20 (95% CI 0.13, 0.27), and 0.14 (95% CI 0.05, 0.24) respectively. After 12 months, the proportion were 0.16 (95% CI 0.08, 0.23), 0.10 (95% CI 0.04, 0.16), and 0.04 (95% CI 0, 0.09) respectively. For HA zone relapse, the RR of HA-CRT versus CRT was 2.72 (95% CI 0.53, 13.87), and for 2-year BM, it was 1.20 (95% CI 0.82, 1.75). Regarding HA-PCI in SCLC, the 1-year BM rate was 0.12 (95% CI 0.07, 0.17), and the 2-year BM rate was 0.20 (95% CI 0.16, 0.25). For HA-WBRT in NSCLC with BM, the 2-year intracranial progression rate was 0.38 (95% CI 0.13, 0.62). There was no significant difference in OS between HA-CRT and CRT.

**Conclusions:**

HA-CRT appears to be safe in lung cancer, but it may not outperform conventional CRT. Larger RCTs comparing HA-CRT and CRT are warranted.

**Systematic review registration:**

https://www.crd.york.ac.uk/prospero/display_record.php?ID=CRD42022360890, identifier CRD42022360890.

## 1 Introduction

Cranial radiation therapy (CRT), which includes prophylactic cranial irradiation (PCI) and whole brain radiotherapy (WBRT), has shown significant efficacy in reducing brain metastasis (BM) and slightly improving overall survival (OS) in patients with lung cancer. BM in lung cancer often present as multiple lesions, making treatment decisions particularly challenging. Patients with lung cancer frequently develop multiple BM, further complicating therapeutic strategies. PCI is a recommended treatment for limited-stage small cell lung cancer (SCLC) but remains controversial for extensive SCLC (1, 2). WBRT remains an important therapeutic option, especially for patients with multiple BM (3). However, long-term neurocognitive dysfunction associated with CRT remains a major concern. The hippocampus, responsible for memory consolidation and cognitive function, is susceptible to radiation-induced damage to neural progenitor cells within it. Consequently, attention has shifted towards CRT with hippocampus avoidance (HA-CRT). However, concerns regarding potential tumor relapse within the hippocampus avoidance (HA) zone and uncertain survival outcomes have impeded its widespread adoption. The effects of HA-CRT on neurocognitive function (NCF), BM, and OS in comparison to conventional CRT are still subjects of debate.

Regarding the impact of HA-CRT on NCF, a prospective study demonstrated encouraging results of HA-PCI on NCF performance in SCLC (4). Additionally, a recent randomized phase III trial suggested a lower rate of NCF impairment in the HA-PCI group (5). However, some researchers have contested the beneficial effects of HA-CRT. A phase II clinical trial evaluating HA-WBRT indicated no significant decrease in NCF scores at 6 months post-treatment (6). A multicenter phase II trial also reported similar effects on NCF between HA-PCI and standard PCI (7). Another randomized phase III trial suggested comparable risks of NCF impairment in patients with SCLC between the two groups, implying that hippocampus sparing may be unnecessary (8). While HA-CRT has been shown to protect against radiation-induced memory loss, it does not prevent deterioration in the executive zone of NCF (9). The debate regarding whether hippocampal sparing increases the risk of BM within the HA zone continues. Some studies have suggested that HA-CRT can elevate the risk of BM in the spared region due to the lower radiation dose received in that area (4, 10, 11). However, other studies have found no significant differences in BM, OS, and quality of life (QoL) between the HA-CRT and standard CRT groups, suggesting that HA-CRT is a safe and feasible alternative (5, 6, 8, 12, 13). Given the absence of published meta-analyses on this topic, the impact of HA-CRT on NCF, BM, and OS in lung cancer patients remains unclear and necessitates further investigation.

To address this knowledge gap, we conducted the first meta-analysis exploring the role of HA-PCI and HA-WBRT in lung cancer. Utilizing data from prospective studies, our study aimed to elucidate the impact of HA-CRT on NCF, BM, and OS in patients with lung cancer.

## 2 Methods

The study was conducted based on the Preferred Reporting Items for Systematic Reviews and Meta-Analysis (PRISMA) guidelines (14) and the Cochrane handbook for Systematic Reviews of Interventions (15). This study was registered in PROSPERO (registration number CRD42022360890, available at: https://www.crd.york.ac.uk/prospero/display_record.php?ID=CRD42022360890).

### 2.1 Search strategy

Online databases including PubMed, Embase, Web of Science, the Cochrane Library, ClincalTrials.gov, Chinese National Knowledge Infrastructure (CNKI), Wanfang, and Weipu (VIP) databases were searched by two of the authors independently for studies update to July 2023. The search terms were (“intracranial” or “brain” or “cranial”) and (“irradiation” or “radiation therapy” or “radiation treatment” or “radiotherapy”), “lung cancer” or “lung carcinoma” or “lung tumor” or “lung neoplasm”, “hippocampus” or “hippocampal”. There was no language restriction.

### 2.2 Selection criteria

Data selection played a crucial role in ensuring the credibility and comprehensiveness of this study. The inclusion criteria for selecting studies were as follows (1): participants diagnosed with lung cancer (2); intervention involving either HA-CRT or conventional CRT (3); evaluation of outcomes related to NCF, BM, and OS. NCF outcomes were assessed based on the decline in total recall (TR), delayed recall (DR), and discrimination scores on the Hopkins Verbal Learning Test—Revised (HVLT-R) or the Free and Cued Selective Reminding Test (FCSRT) at 6 and 12 months post-treatment. TR refers to the total number of items that an individual is able to correctly recall during a learning or memory phase. It represents the comprehensive measure of the individual’s ability to retrieve and reproduce the learned information accurately. TR scores is typically assessed immediately after the learning phase. DR, on the other hand, refers to the number of items that an individual can recall or retrieve after a certain time interval following the completion of the learning or memory task. It provides insight into the individual’s capacity for retaining and retrieving information from long-term memory. Discrimination scores reflect the accuracy of an individual in distinguishing between correct and incorrect responses during learning and memory tasks. They provide a measure of the individual’s ability to accurately discriminate between learned information and unrelated or distractor items. These measures, including TR, DR, and discrimination scores, are commonly utilized in cognitive assessment tools such as the HVLT-R or the FCSRT to evaluate an individual’s memory and cognitive functioning. By examining these measures, we can gain valuable insights into an individual’s learning abilities, memory retention, and accuracy in discriminating relevant information. BM refers to the spread of cancer from its primary site (such as the lungs) to the brain. Diagnosis of brain metastasis typically relies on symptoms and diagnostic procedures like brain magnetic resonance imaging (MRI) or computed tomography scans. OS was defined as the time from randomization to death from any cause (4). Only prospective clinical trials updated until July 2023, were included. Exclusion criteria were applied as follows (1): patients with other diseases or a history of brain radiation therapy (2); insufficient or duplicated data (3); studies categorized as reviews, comments, case reports, observational studies, animal experiments, or other inappropriate study types. In cases where results from the same population were published in different journals, the most complete or recent study was selected. Additionally, randomized controlled trials (RCTs) comparing CRT with or without HA were also included for analysis from the eligible studies mentioned above.

### 2.3 Data collection and quality assessment

The literature was screened according to the pre-established inclusion and exclusion criteria. Initially, two authors conducted a duplicate content check and then proceeded to conduct a thorough review of the abstracts in order to identify and eliminate non-directly relevant articles. Subsequently, they independently reviewed the remaining full-text articles and extracted detailed data from eligible studies. The primary endpoint was NCF outcome. The secondary endpoints were BM and OS. We assessed the quality of non-randomized controlled trials using the Methodological Index for Non-Randomized Studies (MINORS) criteria (16) and that of randomized controlled trials using the Cochrane Collaboration’s tool (17). Any discrepancies were resolved by a third reviewer.

### 2.4 Statistical analysis

For single-arm studies, the proportion of cognitive dysfunction, BM or OS and 95% confidence interval (95% CI) were specifically combined to draw forest plots. For RCTs, risk ratio (RR) and 95% CI were analyzed. We tested statistical heterogeneity using the I^2^ and Cochrane Q test. I^2^ > 50% or p < 0.05 indicates a significant heterogeneity between selected studies, so the random effects model was applied; while I^2^ < 50% or p > 0.05 suggests no significant heterogeneity, then the fixed effects model was performed. To investigate the sources of heterogeneity, we also carried out subgroup analysis in terms of median age (>= 65 vs. < 65 years), proportion of limited stage lung cancer (>= 75% vs. < 75%), median follow-up (>= 24 vs. < 24 months), and radiation therapy techniques (= VMAT or IMRT vs. = TOMO). We performed funnel plots to evaluate publication bias. Meanwhile, we ruled out each single studies in the sensitivity analysis to evaluate the satiability of the results. All analyses were performed by software R 4.1.2 with the meta_v5.2-0 packages.

## 3 Results

### 3.1 Study selection

**Figure 1:**
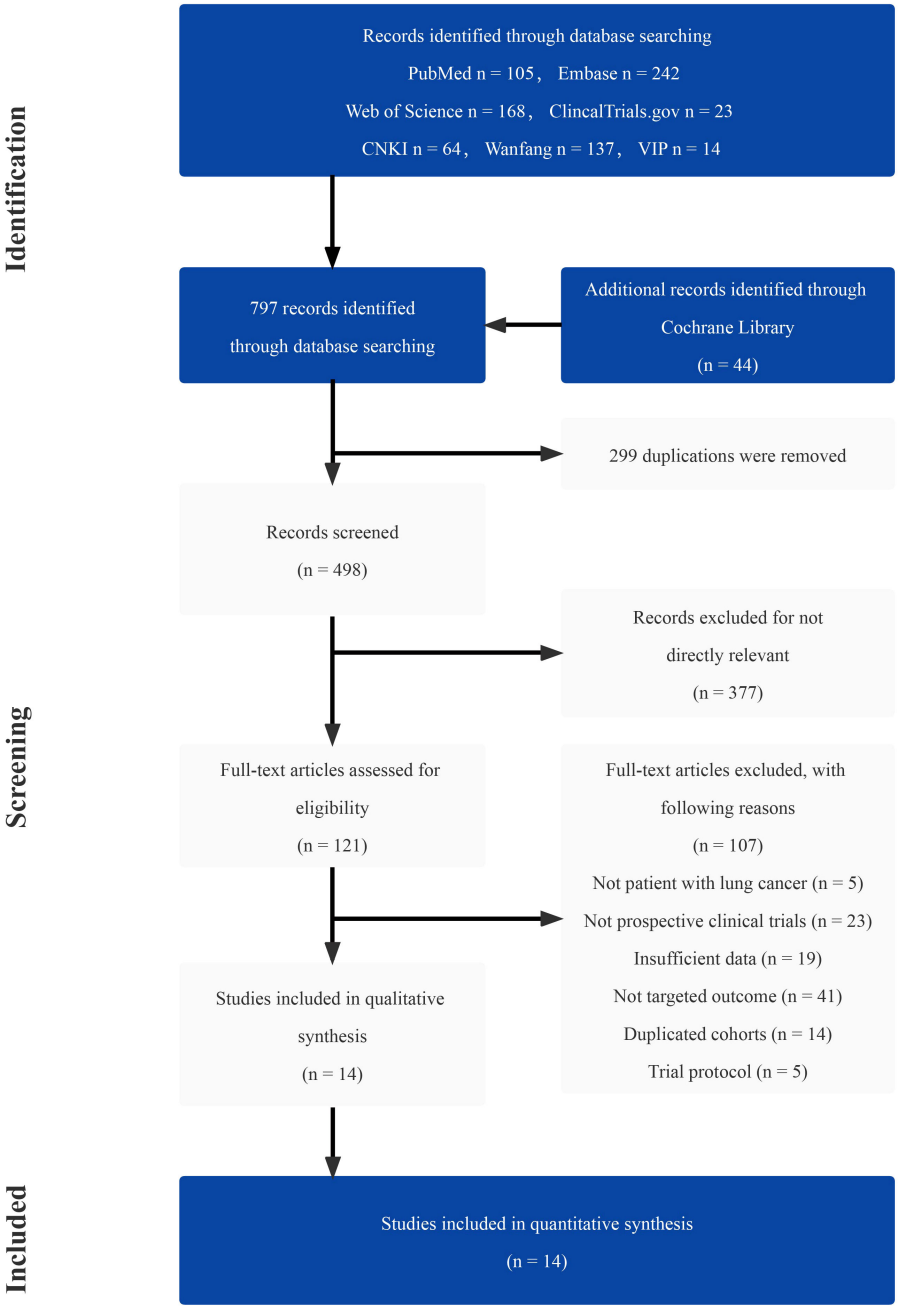
Flow diagram of identifying eligible studies.

A total of 797 studies were screened through the comprehensive search (**Supplementary Table 1**). After reading the title and abstract, 676 of them were excluded due to duplication and irrelevance. The remaining 121 articles were further reviewed, and 107 of them were excluded for the reason of wrong population (n = 5), incorrect study type (n = 23), insufficient data (n = 19), not comparing outcomes of interest (n = 41), duplicated cohorts (n = 14) and trial protocol (n = 5). Ultimately, 14 prospective studies were chosen, including 5 RCTs. The number of eligible patients was 922. The procedure of the literature search is shown in **Figure 1**. The main characteristics of RCTs and selected prospective trials are shown in **Tables 1**, **2**.

**Table 1:** Main characteristics of RCTs included in the meta-analysis.

Author Year	CRT	Patients (Number)		Cancer type	Radiation therapy techniques	Mean hippocampus dose in HA-CRT group (Gy)	Standard CRT schedule (Gy/fraction)
		HA-CRT	CRT				
Rodríguez 2021 (5)	HA-PCI, PCI	75	75	SCLC	IMRT or VMAT	11.6	25/10
Belderbos 2021 (8)		84	84	SCLC	IGRT	< 8.5
Cho 2021 (18)		48	58	LS-SCLC	TOMO	NA
Wang 2021 (19)	HA-WBRT, WBRT	27	20	NSCLC with BM	IMRT or VMAT	NA	30/10
Kong 2020 (20)	HA-PCI, PCI	18	22	LS-SCLC	VMAT	NA	25/10

CRT, cranial radiation therapy; HA-CRT, hippocampal−avoidance cranial radiation therapy; SCLC, small cell lung cancer; LS-SCLC, limited-stage small cell lung cancer; HA-PCI, hippocampal−avoidance prophylactic cranial irradiation; PCI, prophylactic cranial irradiation; ES-SCLC, extensive-stage small cell lung cancer; IMRT, intensity modulated radiation therapy; VMAT, volumetric modulated arc therapy; IGRT, image-guided radiotherapy; TOMO, helical tomotherapy; NA, not available; HA-WBRT, hippocampus-avoidance whole brain radiotherapy; WBRT, whole brain radiotherapy; NSCLC, non-small cell lung cancer.

**Table 2:** Main characteristics of prospective studies included in the meta-analysis.

Author Year	CRT	Patients (number)	Cancer type	Radiation therapy techniques	Mean hippocampus dose in HA-CRT group (Gy)	CRT schedule outside hippocampus (Gy/fraction)
Cook 2021 (12)	HA-PCI	17	SCLC	VMAT	NA	25/10
Zhong 2021 (13)	HA-WBRT	31	NSCLC (54.8%) SCLC (45.2%)	VMAT	< 10	30/10
Corrao 2021 (21)	HA-CRT	150	LC	TOMO	7.6	30/10 or 30/12
Vees 2020 (7)	HA-PCI	42	LS-SCLC	IMRT	NA	25/10
Wang 2019 (6)	HA-WBRT	39	LC	TOMO	< 15	WBRT: 32.4/18 SIB: 48.6/18
Dong 2018 (22)	HA-PCI	49	LS-SCLC (91.8%) ES-SCLC (8.2%)	TOMO	7.23	25/10
Redmond 2017 (4)	HA-PCI	20	LS-SCLC	IMRT	< 8	25/10
Lykkegaard 2016 (23)	HA-PCI	22	LS-SCLC (40.9%) ES-SCLC (59.1%)	IMRT	NA	25/10
Kundapur 2013 (24)	HA-WBRT	39	SCLC	NA	NA	NA

CRT, cranial radiation therapy; HA-CRT, hippocampal−avoidance cranial radiation therapy; SCLC, small cell lung cancer; HA-PCI, hippocampal−avoidance prophylactic cranial irradiation; VMAT, volumetric modulated arc therapy; NA, not available; HA-WBRT, hippocampus-avoidance whole brain radiotherapy; NSCLC, non-small cell lung cancer; LC, lung cancer; TOMO, helical tomotherapy; LS-SCLC, limited-stage small cell lung cancer; IMRT, intensity modulated radiation therapy; WBRT, whole brain radiotherapy; SIB, simultaneous integrated boost; ES-SCLC, extensive-stage small cell lung cancer.

### 3.2 Quality assessment

In general, the quality of selected papers was satisfied. Five RCTs were included and assessed by the Cochrane Collaboration’s tool. As shown in **Supplementary Table 2**, these five trials had high quality. As most of the selected studies were non-randomized trials, the MINORS method was applied, and they were all considered as moderate or high quality (**Supplementary Table 3**). Funnel plots of studies including data of NCF are shown in **Supplementary Figure 5**.

### 3.3 NCF outcomes

**Figure 2:**
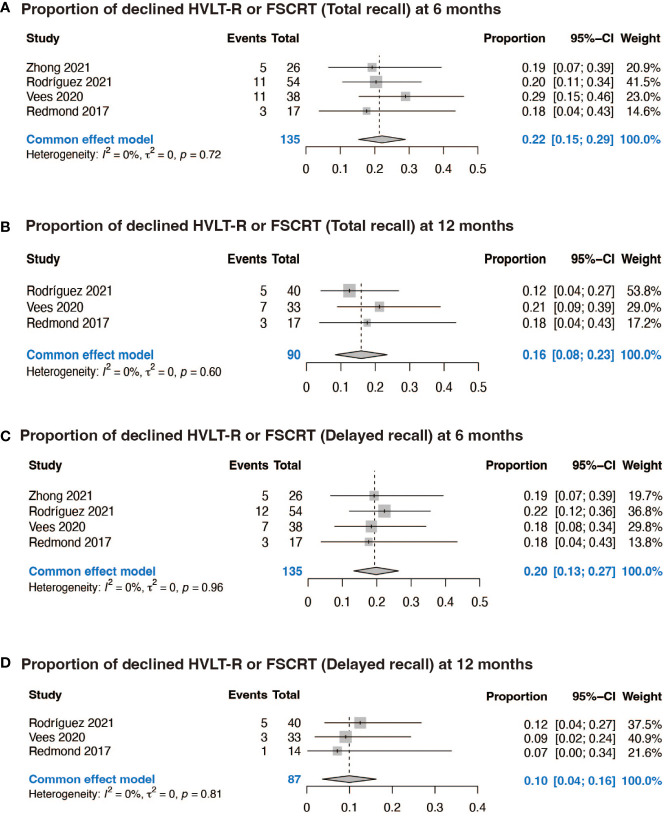
Forest plots of NCF. **(A)** Proportion of declined HVLT-R or FSCRT (Total recall) at 6 months; **(B)** Proportion of declined HVLT-R or FSCRT (Total recall) at 12 months; **(C)** Proportion of declined HVLT-R or FSCRT (Delayed recall) at 6 months; **(D)** Proportion of declined HVLT-R or FSCRT (Delayed recall) at 12 months.

Four prospective studies included specific data of 135 patients with declined HVLT-R or FSCRT TR and DR, and two of them included data of HVLT-R or FSCRT discrimination. At 6 months after neurocognitive tests, the pooled proportion of participants with decline in the performance of HVLT-R or FSCRT TR test was 0.22 (95% CI: 0.15, 0.29, p = 0.72), while at 12 months it was 0.16 (95% CI: 0.08, 0.23, p = 0.60) (**Figures 2A, B**). When it comes to HVLT-R or FSCRT DR, at 6 months the decline rate was 0.20 (95% CI: 0.13, 0.27, p = 0.96), while at 12 months it was 0.10 (95% CI: 0.04, 0.16, p = 0.81) (**Figures 2C, D**). As to HVLT-R or FSCRT discrimination, it was 0.14 (95% CI: 0.05, 0.24, p = 0.68) at 6 months and 0.04 (95% CI: 0, 0.09, p = 0.58) at 12 months (**Supplementary Figure 1**).

### 3.4 BM outcomes

**Figure 3:**
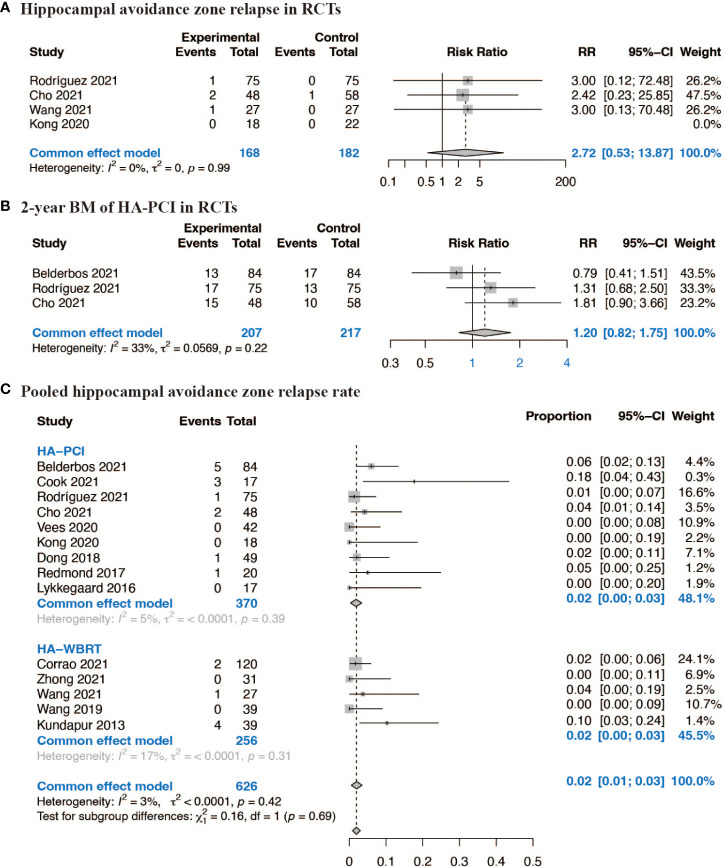
Forest plots of BM. **(A)** Hippocampal avoidance zone relapse in RCTs; **(B)** 2-year BM of HA-PCI in RCTs; **(C)** Pooled hippocampal avoidance zone relapse rate.

In the context of RCTs comparing HA-CRT to traditional CRT, the estimated RR for HA zone relapse was 2.72 (95% CI: 0.53, 13.87, p = 0.99) based on four RCTs, and the estimated RR for 2-year BM was 1.20 (95% CI: 0.82, 1.75, p = 0.22) based on three RCTs, suggesting no significant difference in BM between patients assigned to HA-CRT and traditional CRT (**Figures 3A, B**). The combined proportion of HA zone relapse in all 14 studies was 0.02 (95% CI: 0.01, 0.03, p = 0.42) (**Figure 3C**). The pooled 1-year BM incidence of HA-PCI was 0.12 (95% CI: 0.07, 0.17, p = 0.39), and for 2-year BM, it was 0.20 (95% CI: 0.16, 0.25, p = 0.24). The pooled 2-year intracranial progression of HA-WBRT was 0.38 (95% CI: 0.13, 0.62, p = 0.02) (**Supplementary Figure 2**). Next, we conducted subgroup analyses for HA zone relapse according to median age, proportion of limited stage lung cancer, median follow-up and radiation therapy techniques, and the results were shown in **Supplementary Figure 4**. Subgroup analyses indicated that factors such as median age (>= 65 vs. < 65 years), proportion of limited stage lung cancer (>= 75% vs. < 75%), median follow-up (>= 24 vs. < 24 months), and radiation therapy techniques did not significantly affect the outcomes. To test the satiability, we ruled out each single studies in the sensitivity analysis and the results suggested that the selected studies were reliable (**Supplementary Figure 6**).

### 3.5 OS outcomes

**Figure 4:**
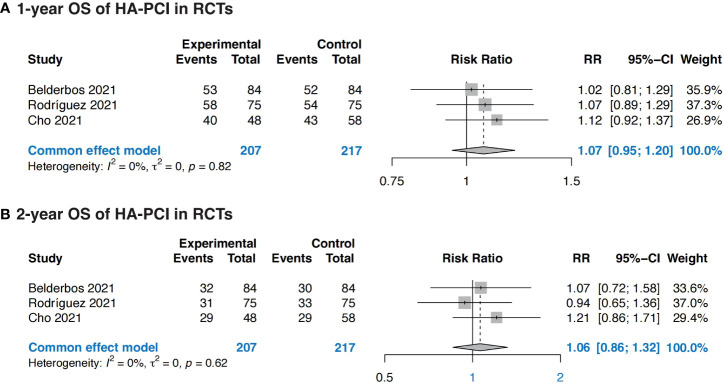
Forest plots of OS. **(A)** 1-year OS of HA-PCI in RCTs; **(B)** 2-year OS of HA-PCI in RCTs.

Three RCTs were eligible for OS outcomes analysis, totally including 207 patients in HA-PCI group and 217 patients in normal PCI group. The combined result revealed no significance in OS in patients with HA-PCI compared to normal PCI, with a RR of 1.07 (95%CI: 0.95, 1.20, p = 0.82) for 1-year OS and 1.06 (95%CI: 0.86, 1.32, p = 0.62) for 2-year OS (**Figure 4**). The pooled 1-year OS of HA-PCI in SCLC was 0.79 (95% CI: 0.71, 0.88, p < 0.01) (**Supplementary Figure 3**).

## 4 Discussion

The efficacy and safety of HA-CRT in patients with lung cancer remain uncertain, and there is ongoing debate in the current research. Our comprehensive analysis indicates that HA-CRT is a safe and feasible option for lung cancer treatment. However, when comparing HA-CRT to conventional CRT, our findings suggest that it may not provide significant improvements in terms of NCF, BM, and OS.

Recent studies have reported conflicting results regarding the impact of HA-CRT on NCF. Some studies have shown positive outcomes, indicating that sparing the hippocampal zone can limit radiation-induced cognitive side effects (4, 5, 22). However, other studies have reported no significant differences in NCF between the HA-CRT group and the CRT group without hippocampal sparing (7, 8). In our meta-analysis, we evaluated the proportion of patients experiencing declines in cognitive test performance. However, due to variations in observation times for outcome indicators, direct comparison and summarization were challenging. Our analysis did not demonstrate a significant decline in cognitive function at 6 and 12 months after early HA-CRT.

BM is a significant cause of treatment failure in lung cancer, typically occurring within 2 years of diagnosis. CRT has been shown to reduce BM and slightly improve OS in lung cancer patients. Consequently, concerns regarding cancer relapse in the brain, particularly in the lower-dose hippocampal zone, have emerged with HA-CRT. Nevertheless, our study indicates that HA-CRT is a safe and feasible alternative to traditional CRT, as it does not increase the risk of BM even in the lower-dose region. However, it does not provide significant improvements in OS for lung cancer patients.

Elderly patients aged 65 years and above pose unique challenges in treatment, as standard approaches are often associated with significant survival benefits but also toxicities and side effects. Previous studies have demonstrated the detrimental effects of radiation on brain tissue, particularly the hippocampus (25). In aged mice, WBRT has been shown to result in memory impairment, abnormal behavior, and decreased stress response (26). Clinical studies have reported radiation encephalopathy and dementia as sequelae of cranial irradiation in elderly patients (27). Although our subgroup analysis based on median age did not yield significant results, likely due to limited data, further RCTs comparing HA-CRT and CRT are eagerly awaited to explore potential differences.

The conflicting results observed in existing studies may be attributed to differences in radiotherapy techniques. Techniques such as intensity-modulated radiation therapy (IMRT), helical tomotherapy (TOMO), volumetric modulated arc therapy (VMAT), and image-guided radiotherapy (IGRT) can achieve selective hippocampal protection without compromising the tumor target dose. Comparisons between TOMO and linac-based IMRT have demonstrated greater hippocampal protection with TOMO in terms of average dose parameters (11). VMAT and linac-based IMRT have been found to provide adequate control of hippocampal dose, with VMAT offering better dose uniformity (28). Additionally, variations in hippocampal delineation techniques across studies can contribute to differences in outcomes. Accurate delineation of the hippocampus is crucial and may impact the final results (29). Furthermore, studies have employed different neurocognitive evaluation scales and follow-up durations, which may contribute to variability in outcomes. Therefore, further research with larger trials and longer follow-up periods is needed to explore potentially smaller differences between HA-CRT and conventional CRT.

Furthermore, in the context of evolving treatment strategies for lung cancer patients with BM, another noteworthy trend has emerged. Clinicians are increasingly embracing a comprehensive approach that involves the application of stereotactic radiation therapy (SRT) to address all metastatic lesions, accompanied by vigilant monitoring through MRI. While our meta-analysis primarily focused on the specific aspect of HA-CRT, we acknowledge the significance of this broader approach. SRT, with its precision and ability to target individual metastatic lesions, is gaining recognition for its potential to achieve effective local control while preserving healthy brain tissue. The frequent use of MRI follow-up protocols facilitates the early detection of new metastases or changes in existing lesions, enabling timely intervention when required. This comprehensive strategy represents a promising avenue for enhancing patient care. Our analysis underscores the importance of ongoing research and exploration into these evolving treatment paradigms, which hold the potential to impact neurocognitive function, brain metastasis control, and overall survival in lung cancer patients.

To our knowledge, this study represents the first meta-analysis examining the role of HA-CRT in patients with lung cancer. Nevertheless, it is essential to acknowledge the limitations of our study. Firstly, HA-CRT is a relatively new approach, and the number of studies comparing HA-CRT and conventional CRT was limited. We were only able to identify 17 prospective studies, including 5 RCTs, with most of the selected studies being single-arm trials. As a result, while we provided the latest pooled data on NCF, BM and OS, we were unable to definitively assess the superiority of HA-CRT over CRT in terms of NCF, primarily due to the scarcity of relevant data. Secondly, the included trials utilized potentially different methods for delineating the hippocampus and various radiation therapy techniques, which might have introduced some degree of bias into our analysis. To improve the comparability of future studies, standardization of these approaches would be beneficial. Moreover, it is imperative to stress the critical need for standardized dose constraints in future studies within this field. The absence of such standardization in current literature poses significant challenges when comparing trial outcomes and deriving conclusive insights. To enhance the comparability and robustness of forthcoming research, it is essential for the radiation oncology community to collectively establish and adopt universally recognized dose constraints for HA-CRT. Additionally, it is worth noting that while some research suggests that Memantine, by modulating the activity of N-methyl-D-aspartic acid receptors, may reduce neuronal damage and inflammation induced by radiation therapy, our meta-analysis did not include relevant studies on Memantine’s impact. This is an area that warrants further investigation and could potentially enhance our understanding of HA-CRT and its effects on the hippocampus. Despite these limitations, our meta-analysis has provided valuable and objective information on the use of HA-CRT in lung cancer. It offers insights that can guide further research and clinical decision-making in this area.

## 5 Conclusion

In summary, our findings reveal that HA-CRT in lung cancer is safe and feasible, but it may not perform much better than conventional CRT no matter in NCF, BM, and OS. However, further studies with longer follow-up and more RCTs comparing HA-CRT and CRT in lung cancer are still worth looking forward to.

## Data availability statement

The original contributions presented in the study are included in the article/**Supplementary Material**. Further inquiries can be directed to the corresponding author.

## Author contributions

YZ: Conceptualization, Data curation, Formal Analysis, Investigation, Methodology, Writing – original draft, Writing – review & editing. LY: Formal Analysis, Methodology, Writing – original draft. BF: Investigation, Methodology, Software, Writing – original draft. MT: Data curation, Supervision, Validation, Writing – review & editing. FN: Conceptualization, Data curation, Formal Analysis, Funding acquisition, Investigation, Writing – review & editing, Project administration.
