# Supplementary material for: Cranial radiation therapy with hippocampus avoidance in lung cancer treatment: systematic review and meta-analysis
Source: Front Oncol. 2023 Oct 23;13:1268754. doi: 10.3389/fonc.2023.1268754 (PMC10626474; doi:10.3389/fonc.2023.1268754)
Supplement: Supplementary file 1 [file DataSheet_1.docx]

Supplementary Material

**Supplementary Table 1** Search strategies of English databases

**Supplementary Table 2** Quality assessment for RCTs though the Cochrane Collaboration’s tool

**Supplementary Table 3** Quality assessment for non-randomized studies using Methodological Index for Non-Randomized Studies criteria

**Supplementary Figure 1** Pooled analysis and subgroup analysis of NCF

A. Proportion of declined HVLT-R or FSCRT (Discrimination) at 6 months; B. Proportion of declined HVLT-R or FSCRT (Discrimination) at 12 months

**Supplementary Figure 2** Pooled analysis of BM

A. 1-year BM of HA-PCI; B. 2-year BM of HA-PCI; C. 2-year intracranial progression in HA-WBRT

**Supplementary Figure 3** Pooled 1-year OS of HA-PCI

**Supplementary Figure 4** Subgroup analysis of HA zone relapse

A. Subgroup analysis according to median age; B. Subgroup analysis according to proportion of limited-stage lung cancer; C. Subgroup analysis according to median follow-up; D. Subgroup analysis according to radiation therapy techniques

**Supplementary Figure 5** Funnel plots of NCF

**Supplementary Figure 6** Sensitivity analysis for RCTs included

A. Hippocampal avoidance zone relapse in RCTs; B 2-year BM of HA-PCI in RCTs; C. 1-year OS of HA-PCI in RCTs; D. 2-year OS of HA-PCI in RCTs

**Supplementary Table 1** Search strategies of English databases

| Database | Keywords | |
| --- | --- | --- |
| PubMed | #1 | ("intracranial"[All Fields] OR "brain"[All Fields]) AND ("irradiation"[All Fields] OR "radiation therapy"[All Fields] OR "radiation treatment"[All Fields] OR "radiotherapy"[All Fields]) |
|  | #2 | ("lung cancer"[All Fields] OR " lung carcinoma"[All Fields] OR " lung tumor"[All Fields] OR "lung neoplasm"[All Fields] |
|  | #3 | ("hippocampus"[All Fields] OR "hippocampal"[All Fields]) |
|  | #4 | #1 AND #2 AND #3 |
| Embase | #1 | irradiation OR (radiation AND therapy) OR (radiation AND treatment) OR radiotherapy |
|  | #2 | (lung AND cancer) OR (lung AND carcinoma) OR (lung AND tumor) OR (lung AND neoplasm) |
|  | #3 | hippocampus OR hippocampal |
|  | #4 | #1 AND #2 AND #3 |
| Wed of Science | #1 | (“intracranial” or “brain”) and (“irradiation” or “radiation therapy” or “radiation treatment” or “radiotherapy”) (All Fields) |
|  | #2 | “lung cancer” or “lung carcinoma” or “lung tumor” or “lung neoplasm” (All Fields) |
|  | #3 | “hippocampus” or “hippocampal” (All Fields) |
|  | #4 | #1 AND #2 AND #3 |
| Cochrane Library | #1 | (“intracranial” or “brain”) and (“irradiation” or “radiation therapy” or “radiation treatment” or “radiotherapy”) in All Text - (Word variations have been searched) |
|  | #2 | “lung cancer” or “lung carcinoma” or “lung tumor” or “lung neoplasm” in All Text - (Word variations have been searched) |
|  | #3 | “hippocampus” or “hippocampal” in All Text - (Word variations have been searched) |
|  | #4 | #1 AND #2 AND #3 |
| Clincaltrials.gov | #1 | hippocampus or hippocampal |
|  | #2 | Lung Cancer |
|  | #3 | #1 \| #2 |

**Supplementary Table 2** Quality assessment for RCTs though the Cochrane Collaboration’s tool

| Author Year | Randomization | Allocation concealment | Blinding of participants and personnel | Blinding of outcome assessment | Incomplete outcome data | Selective reporting | Other bias |
| --- | --- | --- | --- | --- | --- | --- | --- |
| Rodríguez 2021 | Low risk | Low risk | Low risk | Low risk | Low risk | Low risk | Low risk |
| Belderbos 2021 | Low risk | Low risk | Low risk | Low risk | Low risk | Low risk | Low risk |
| Cho 2021 | Unclear | Low risk | Unclear | Low risk | Low risk | Low risk | Low risk |
| Wang 2021 | Unclear | Low risk | Unclear | Low risk | Low risk | Low risk | Low risk |
| Kong 2020 | Low risk | Unclear | Unclear | Unclear | Low risk | Low risk | Low risk |

**Supplementary Table 3** Quality assessment for non-randomized studies using Methodological Index for Non-Randomized Studies criteria

| Criteria for assessment | Cook 2021 | Zhong 2021 | Corrao 2021 | Vees 2020 | Wang 2019 | Dong 2018 | Redmond 2017 | Lykkegaard 2016 | Kundapur 2013 |
| --- | --- | --- | --- | --- | --- | --- | --- | --- | --- |
| Clearly stated aim | 2 | 2 | 2 | 2 | 2 | 2 | 2 | 2 | 2 |
| Inclusion of consecutive patients | 2 | 2 | 2 | 2 | 2 | 2 | 2 | 2 | 2 |
| Prospective data collection | 2 | 2 | 2 | 2 | 2 | 2 | 2 | 2 | 2 |
| Endpoints appropriate of study | 2 | 2 | 2 | 2 | 2 | 2 | 2 | 2 | 2 |
| Unbiased assessment of study endpoint | 1 | 2 | 1 | 1 | 0 | 0 | 1 | 0 | 1 |
| Follow-up period appropriate to study aim | 2 | 0 | 2 | 2 | 2 | 2 | 2 | 0 | 2 |
| Loss to follow up less than 5% | 2 | 2 | 2 | 2 | 2 | 2 | 2 | 2 | 2 |
| Prospective calculation of the study size | 0 | 0 | 0 | 0 | 0 | 0 | 2 | 0 | 0 |
| Adequate control group | * | * | * | * | * | * | * | * | * |
| Contemporary groups | * | * | * | * | * | * | * | * | * |
| Baseline equivalence of groups | * | * | * | * | * | * | * | * | * |
| Adequate statistical analysis | * | * | * | * | * | * | * | * | * |
| Total score | 13 | 12 | 13 | 13 | 12 | 12 | 15 | 10 | 13 |

**Supplementary Figure 1** Pooled analysis and subgroup analysis of NCF

A. Proportion of declined HVLT-R or FSCRT (Discrimination) at 6 months; B. Proportion of declined HVLT-R or FSCRT (Discrimination) at 12 months

**
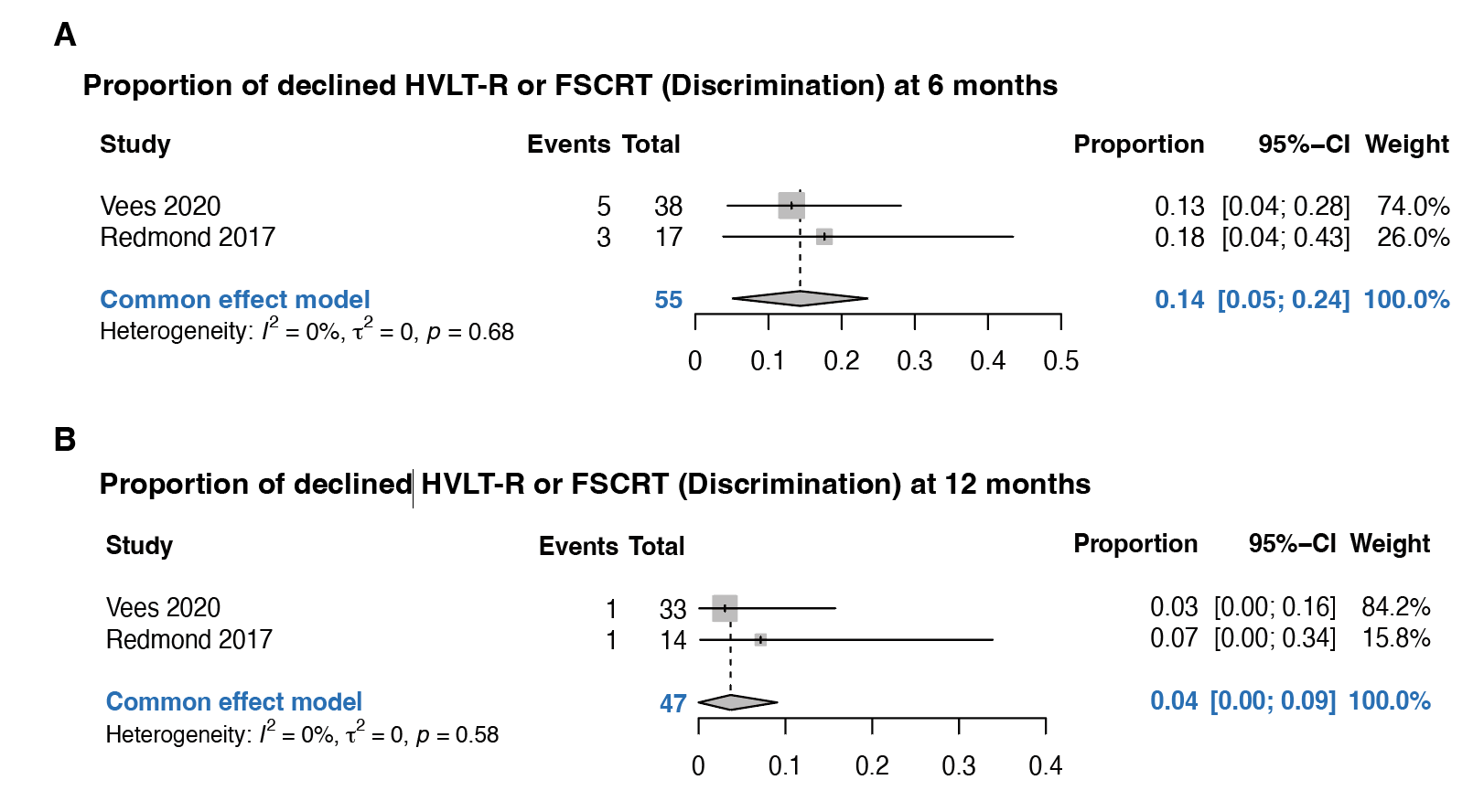
**

**Supplementary Figure 2** Pooled analysis of BM

A. 1-year BM of HA-PCI; B. 2-year BM of HA-PCI; C. 2-year intracranial progression in HA-WBRT

**
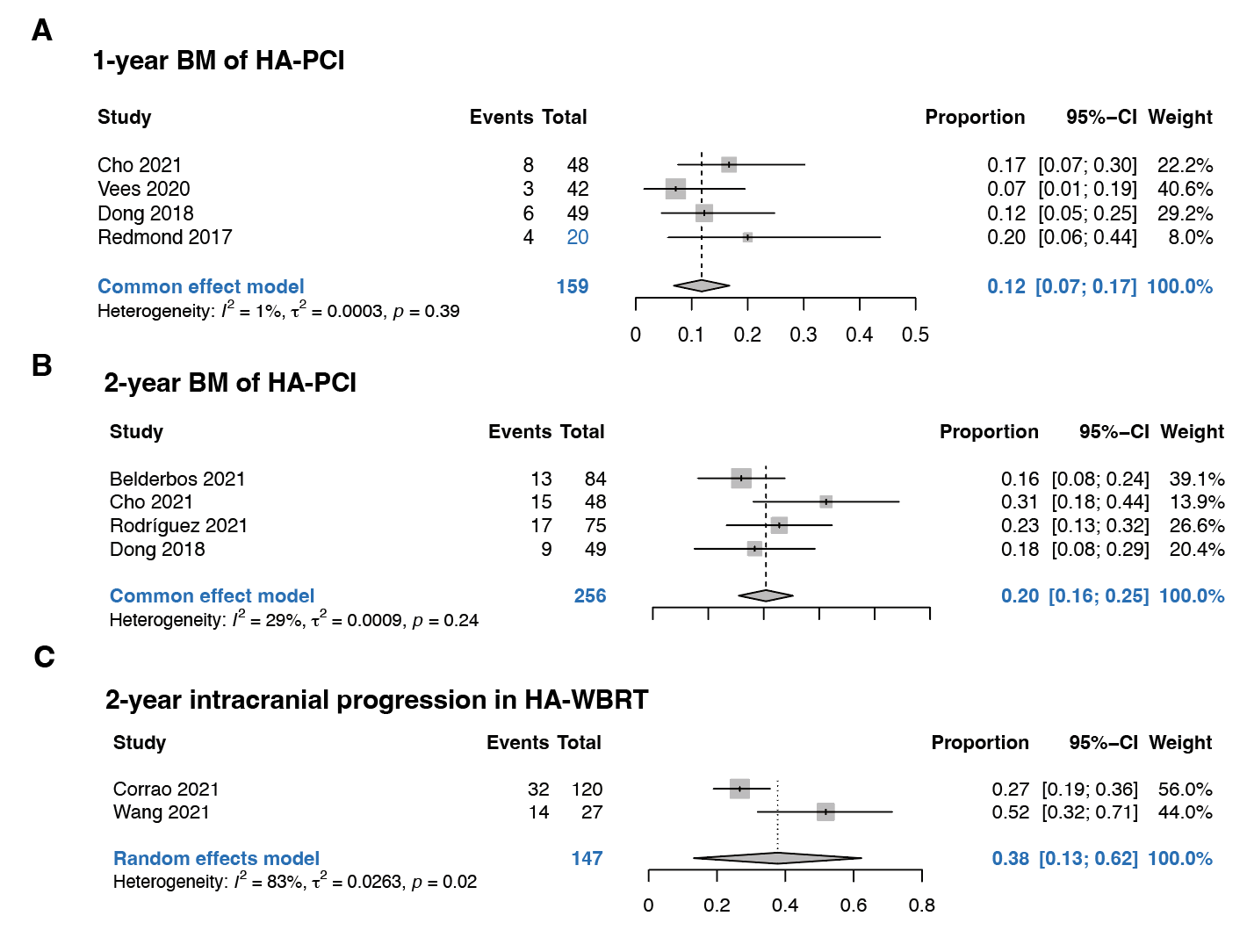
**

**Supplementary Figure 3** Pooled 1-year OS of HA-PCI

**
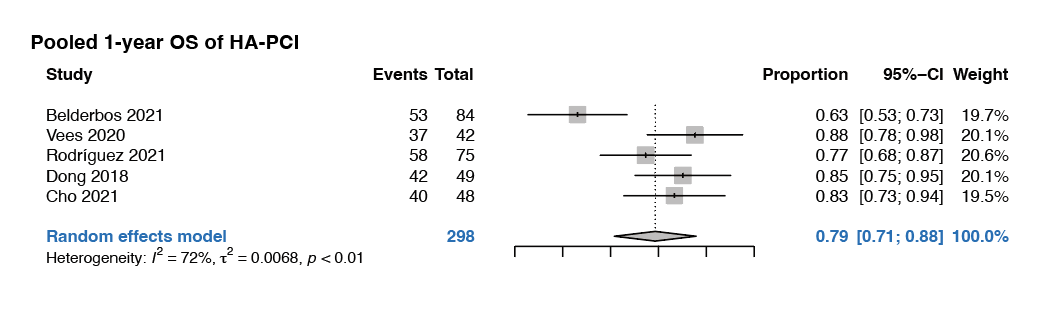
**

**Supplementary Figure 4** Subgroup analysis for HA zone relapse

**
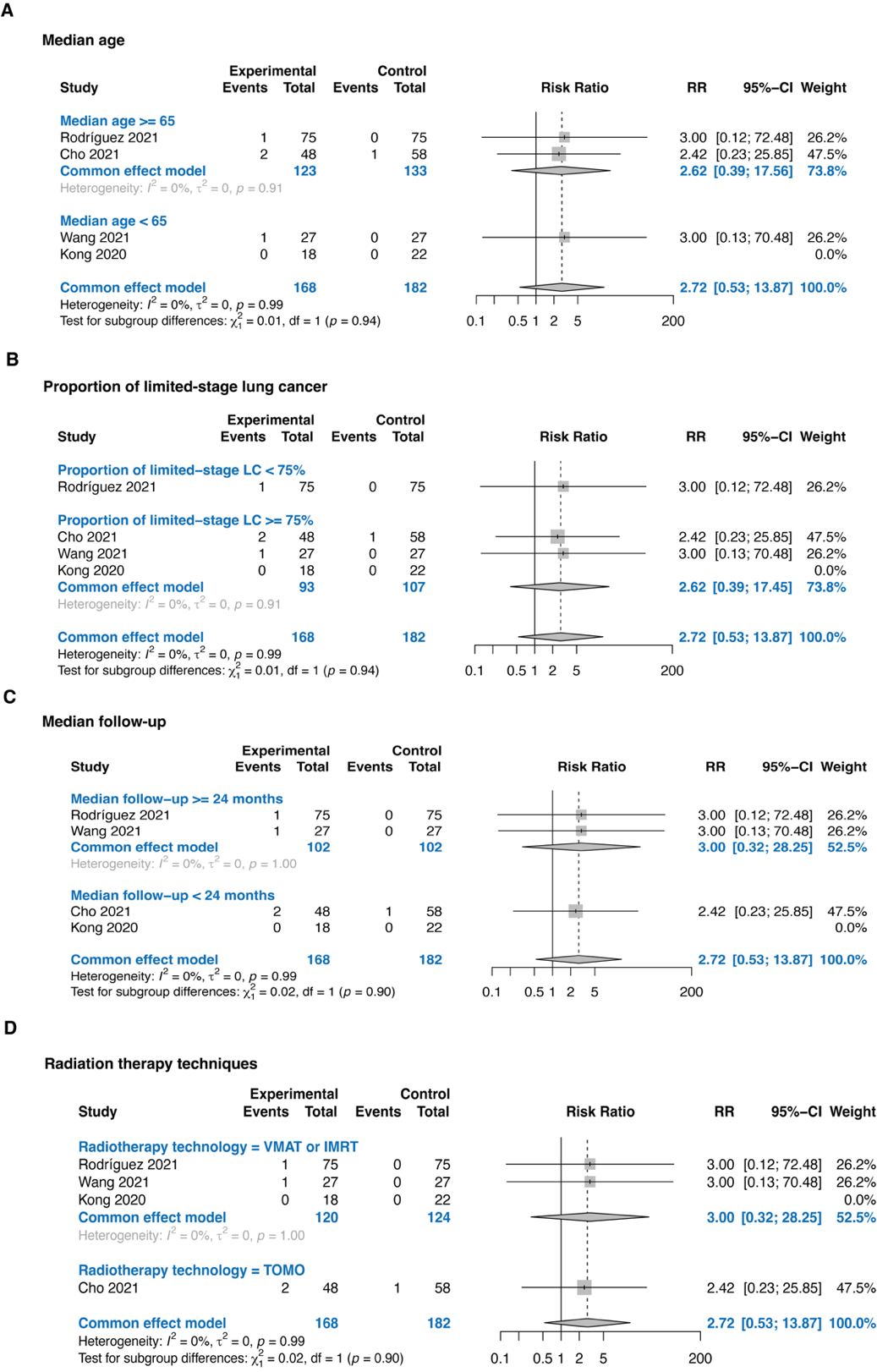
**A. Subgroup analysis according to median age; B. Subgroup analysis according to proportion of limited-stage lung cancer; C. Subgroup analysis according to median follow-up; D. Subgroup analysis according to radiation therapy techniques

**Supplementary Figure 5** Funnel plots of NCF


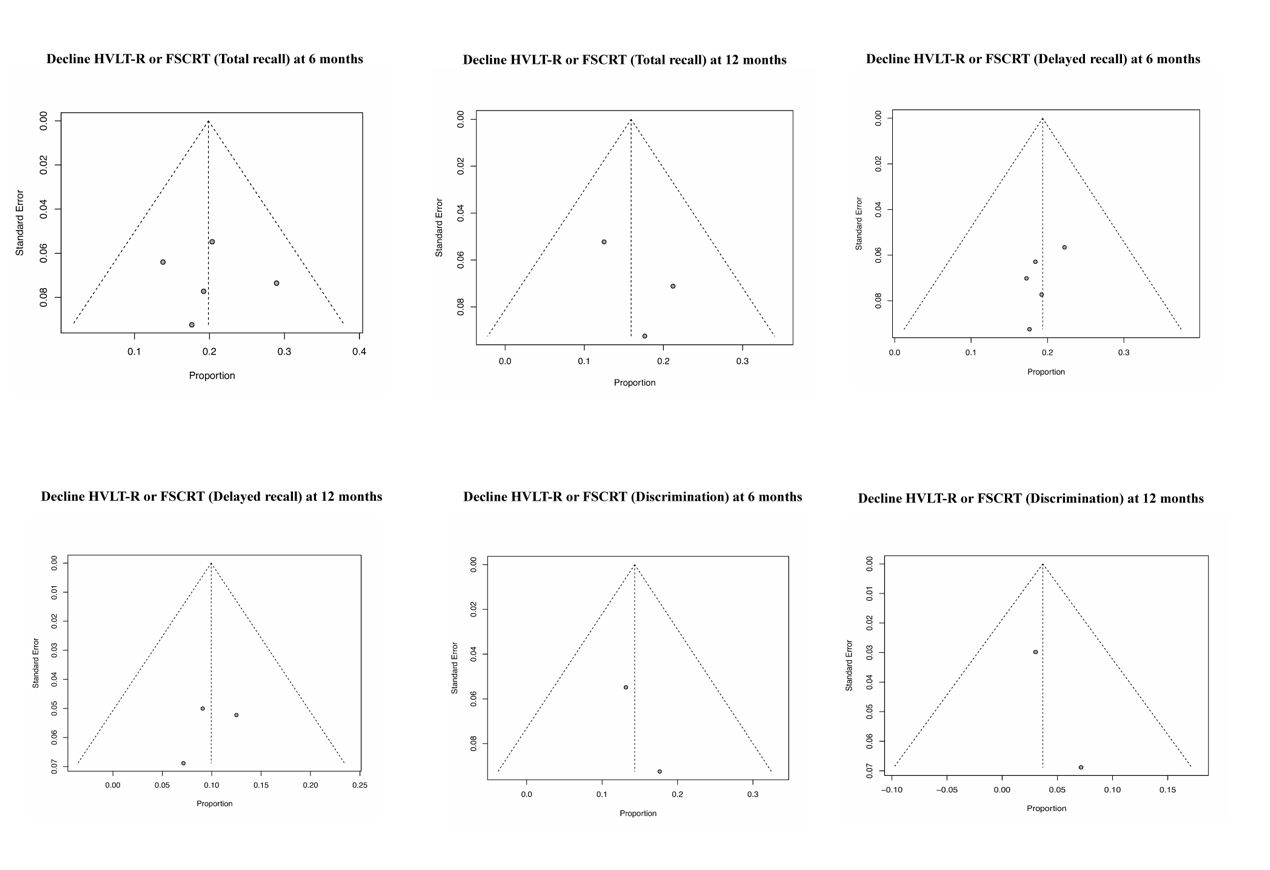


**Supplementary Figure 6** Sensitivity analysis for RCTs included

A. Hippocampal avoidance zone relapse in RCTs; B 2-year BM of HA-PCI in RCTs; C. 1-year OS of HA-PCI in RCTs; D. 2-year OS of HA-PCI in RCTs

**
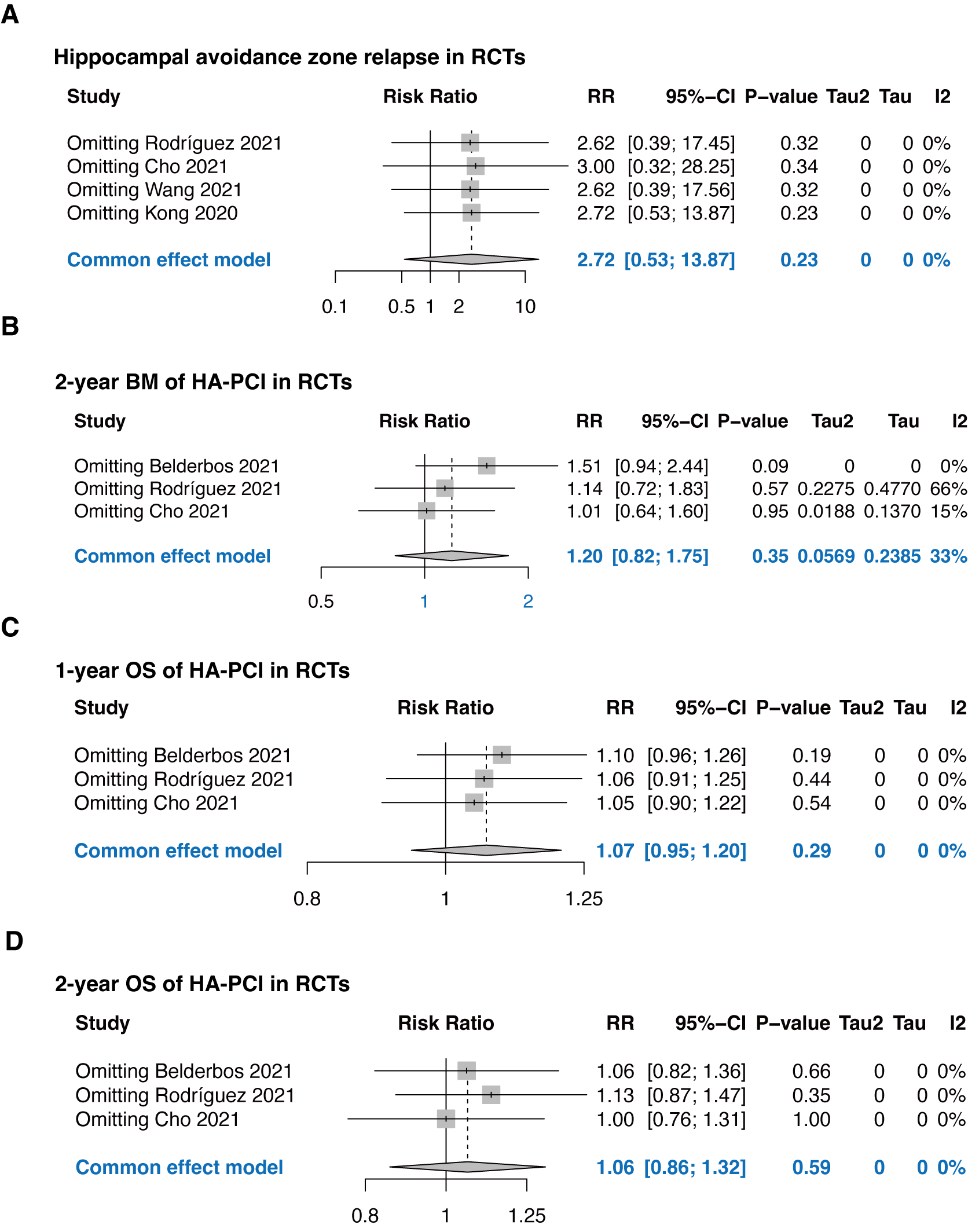
**
